# Practices of Breast Self-Examination and Associated Factors among Female Debre Berhan University Students

**DOI:** 10.1155/2017/8026297

**Published:** 2017-05-17

**Authors:** Kalayu Birhane, Miskir Alemayehu, Belayneh Anawte, Gebru Gebremariyam, Ruth Daniel, Semeneh Addis, Teshome Worke, Abdurrahman Mohammed, Wassie Negash

**Affiliations:** Department of Public Health, College of Health Science, Debre Berhan University, Debre Berhan, Ethiopia

## Abstract

**Background:**

Breast cancer is the most prevalent form of cancer in Ethiopia of all female cancers. It is considered to be a progressive disease with a poor prognosis if detected late. Breast self-examination is an important prevention method of breast cancer. This study was aimed at assessing practice and associated factors of breast self-examination (BSE) among female Debre Berhan University students in Ethiopia.

**Methods:**

A cross-sectional study was conducted in 2015 among 420 using self-administrated questionnaire. Multistage sampling technique was used to select the study participants. Bivariate and multivariate logistic regression analysis were done.

**Results:**

Majority of the study participants, 338 (84.5%), were between 20 and 24 years old with the mean age of 21.1 ± 1.65. Only 14 (3.5%) had family history of breast cancer. Two hundred fifty-six (64%) of the participants had heard about BSE and 30.25% had good knowledge about BSE. Mass media were the most common source of information about breast cancer. Few of the participants (28.3%) had performed BSE. Lack of knowledge on how to perform BSE was cited as the main reason for not practicing BSE. Knowing how to perform, when to perform, and position to perform BSE and having a perception that BSE is important and useful to detect breast cancer were significant predictors of practices of BSE.

**Conclusions:**

This study revealed that most of the participants had low knowledge and practice of BSE. Therefore, it important to develop health educational programs in the university to raise awareness about BSE and breast cancer so as to practice self-breast examination.

## 1. Introduction

Breast cancer is characterised by the uncontrolled growth of abnormal cells in the milk producing glands of the breast or in the passages (ducts) that deliver milk to the nipples [1]. Breast cancer is the most common cancer and the leading cause of cancer death for women which accounts for 23% of all female cancers globally [2]. Worldwide, an estimated 1.7 million women were diagnosed with breast cancer and about 522,000 women died from breast cancer in 2012. Between 2008 and 2012, breast cancer incidence rate has increased by more than 20%, while mortality has increased by 14%. Breast cancer is also a leading cause of cancer death in the less developed countries of the world [3]. For women aged 15–49 years, twice as many breast cancer cases are diagnosed in developing countries than in developed countries [2]. In low-resource settings, 7 out of 10 people newly diagnosed with breast cancer die while 2 out of 10 die in high-resource settings [4]. In Ethiopia, breast cancer is the first leading cancer among females with 24.4% prevalence rate. In 2014, 12,956 women were diagnosed with breast cancer and 26,200 women died by breast cancer [5].

Breast cancer is a public health problem. It attacks women in their most productive years of life but breast cancer can be cured with limited resources if detected early, but treating advanced stage disease is expensive and outcome is often poor [6]. The most important strategies for achieving early detection of breast cancer are mammography and physical examination of the breasts by a physician or qualified health workers or clinical breast examination (CBE) and breast self-examination (BSE). BSE is a process whereby women examine their breasts regularly to detect any abnormal swelling or lumps in order to seek prompt medical attention [7]. While mammography helps to detect breast cancer before women feel a lump, breast self-examination also helps women to be familiar with how their breast look and feel so they can alert their health care professionals if there is any change. Johns Hopkins Medical center states, “Forty percent of diagnosed breast cancers are detected by women who feel a lump, so establishing a regular breast self-exam is very important” [8]. Breast self-examination, carried out once monthly, between the 7th and 10th day of the menstrual cycle, goes a long way in detecting breast cancer at the early stages of growth when there is low risk of spread, ensuring a better prognosis when treated [7]. Early diagnosis has a positive effect on the prognosis and limits the development of complications and disability. Furthermore, it increases life quality and survival [9].

As many studies indicated the practices of BSE are low among university students. A study conducted in Cameroon among female undergraduate students in the University of Buea indicated that only 9.0% knew how to perform BSE, only 13.9% knew what to look for while performing BSE, and only 3% had performed BSE regularly. Furthermore, Lack of knowledge on BSE was cited as the main reason for not performing BSE [10]. Similarly, another study conducted in Nigeria has also shown that only 19.0% of the study participants were performing BSE monthly [11].

Limited knowledge about the realities of breast cancer and lack of knowledge about the importance of self-examination and how it is performed are the main barriers for not practicing BSE. However, the magnitude practices of BSE are limited in our country, particularly in the study area. Therefore, this study aimed to assess the magnitude of practices of BSE and associated factors among female DBU students.

## 2. Methodology

### 2.1. Study Design and Setting

Institutional based cross-sectional study design was conducted on January 2015 in Debre Berhan University, which was established in 2007. The university is found in Debre Berhan Town, the capital city of North Showa Administrative Zone of Amhara Regional State. It has ten colleges with a total of 10551 regular undergraduate students. Of this 3959 are female students. In the town, there are three health centers and one government and one private hospitals.

All regular undergraduate female students were included in the study. However, students who live outside the university (nondormitory) were excluded.

### 2.2. Sample Size Determination and Sampling Procedures

The sample size (420) was calculated using single population proportion formula by Epi Info software based on the following assumption: 22.7% of university students who practice breast self-examination [12], 95% CI, 5% marginal error, 3959 number of undergraduate female students, 1.5 design effect, and 10% of nonresponse rate. All female regular DBU students were included in the study. Since health science students had some information about breast cancer from their courses, they were excluded from the study. Multistage sampling technique was used in the study. In first stage, seven blocks of female dormitory were randomly selected and the calculated sample size was allocated to each selected block proportional to size. In the second stage, study participants were selected from each block using simple random sampling proportion to size.

### 2.3. Data Collection Procedure

A self-administrated questionnaire was used to collect data. The questionnaire was initially prepared in English by reviewing different literature [12–14] and then translated into Amharic and backtranslated into English. The questionnaire comprises four sections: sociodemographic characteristics, knowledge, attitude, and practices of BSE questions. Study participants were given printed copies of the questionnaire and allowed time to fill their response at their will and convenience and in a private, confidential setting. Participant then returned these questionnaires anonymously. A pretest study was carried out upon twenty female students to detect difficulties that may arise during the study and to estimate the required time to fill the questionnaire. Some modification was made on the questionnaire after the pretest.

### 2.4. Measurement

Participant's knowledge towards BSE was measured by the total number of correct answers to 9 items on knowledge questions. A knowledge score was calculated for each participant based on the number of questions correctly answered in the knowledge section. A score of “1” was assigned to every correct answer and a score of “0” to incorrect responses. Knowledge questions were scored and pulled together. The overall knowledge score of the participants were categorized into three: poor, medium, and good knowledge. Participants who answer less than 50% of the knowledge questions were considered as having poor knowledge. Similarly, participants who answer 50–75% and greater than 75% were also classified as having medium and good knowledge about BSE, respectively [15]. Furthermore, participant's practices of BSE were determined from binary outcome variable (yes, no).

### 2.5. Data Processing and Analysis

After checking its completeness, the collected data was entered in Epi Info version 3.5.2 and then exported in to Statistical Package for Social Science (SPPS) version 20 for analysis. Frequency, mean, standard deviation, and percentages were used for descriptive statistics. Multivariable logistic regression analyses were used to identify independent predictors of BSE. Adjusted odds ratio (AOR) with 95% CI was used to measure the strength of association. Statistical significance was set at *P* < 0.05.

### 2.6. Ethics Approval and Consent to Participate

Ethical clearance was obtained from Debre Berhan University, College of Health Science Ethical Committee (chs/cbe/2/07). The data was collected after clear discussion with the study participants about the purpose and the procedures of the study and after obtaining informed consent from each respondent. Participants were assured that they would never face any problem for participating in the study. Privacy and confidentiality of the study participants were strictly maintained.

## 3. Results

### 3.1. Sociodemographic Characteristics of the Study Participants

A total of 420 female DBU students filled the questionnaire correctly which gives response rate of 94%. Most of the participant students (84.5%) were between 20 and 24 age categories with mean age of 21.1 ± 1.65 and single by marital status. Of the participants, 14 (3.5%) had family history of breast cancer (Table 1).

### 3.2. Knowledge of Breast Self-Examination among Female DBU Students

Two hundred fifty-six (64%) of the participants had heard of BSE and 143 (35.8%) of the participants knew how to perform BSE. Furthermore, 3 in 10 (30.5%) participants were aware when to perform BSE. The main sources of information were mass media (39.8%) and health professions (22.3%). Overall, 37.3%, 32.5%, and 30.25% of the study participants had poor (<50%), medium (50–75%), and good knowledge (>75) on BSE, respectively (Table 2).

### 3.3. Attitude towards BSE among Female DBU Students

Almost all of the study participants approved that BSE is important and useful to detect breast cancer and 89.9% of the participants also believed that early detection will increase the chance of long term survival. Moreover, 93% of the participant students believe that BSE is not out of the community social norm. Almost all (96.8%) of the study participants stated that they will go to health facilities if they had any symptoms of breast cancer (Table 3).

### 3.4. Practices of Breast Self-Examination among Female DBU Students

**Figure 1:**
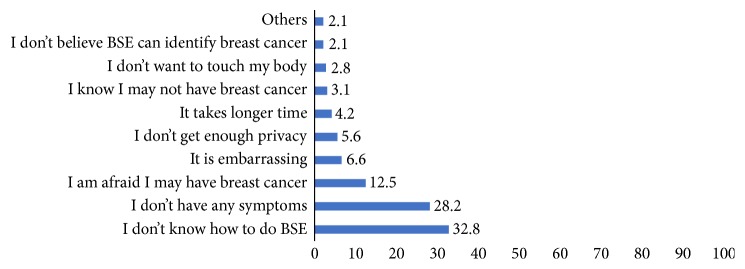
The reason for not performing BSE among Female DBU students.

Only 113 (28.3%) of the participants had ever performed BSE one year preceding the study. Among the participants who practiced BSE 61.9% were practicing monthly. The main reason cited by participants for performing BSE was to know early if they have any change on their breast (64.6%). However, majority (71.7%) of the participants were not practicing BSE. The main reasons for not performing BSE were lack of knowledge on how to conduct BSE and not having any symptoms of breast cancer (Figure 1).

### 3.5. Factors Associated with Practicing of BSE among Female DBU Students

All the independent factors were checked for having an association with practicing of BSE in bivariate logistic regression. College, year of study, having heard about breast cancer, knowing how to perform BSE, knowing when to perform BSE, knowing the three positions to perform BSE, and believing that BSE is important and useful to detect breast cancer were shown as having association with practicing of BSE. However, after controlling the confounding factors, variables remained as predictor factors of practicing BSE were knowing how to perform BSE [AOR = 11.2, 95% CI (4.542–27.607)], knowing when to perform BSE [AOR = 3.5, 95% CI (1.620–7.593)], knowing the three positions to perform BSE [AOR = 2.3, 95% CI (1.104–4.599)], and believing that BSE is important and useful to detect breast cancer [AOR = 6.8, 95% CI (1.640–28.509)] (Table 4).

## 4. Discussion

Breast cancer is the most common cancer among women worldwide. The present study was conducted to determine the practice of BSE and associated factors among female DBU students.

The result of the current study revealed that only 28.3% of the participants had performed BSE. This finding is almost similar to the results of study conducted in Nigeria [11] and Malaysia [13] but inconsistent with the study findings among female university students in Yemen [14], Ajman, United Arab Emirate (UAE) [12], University of Buea Cameroon [10], and Madawalabu University Ethiopia [16]. The reasons for this inconsistency might be due to the difference in the level of knowledge towards BSE among the university students. The other possible explanation for this difference may be due to the difference in accessibility to information or mass media and the emphasis given to BSE. Similar to the studies from Ethiopia [16], Nigeria [11], Cameroon [10], Yemen [14], UAE [12], and Malaysia [13] in this study, the main reasons for not practicing BSE were “lack of knowledge on how to perform (32.8%)” and “not having any symptoms of breast cancer (28.2%).” This might be due to inadequate health education awareness programs to this target population.

Despite the fact that 64% of the participants had heard about BSE, significant proportion of the participants had limited knowledge about BSE. Almost only 3 in 10 participants knew how to perform and when to perform BSE, respectively, which is similar to finding from Malaysia [13] but higher than the result from a study conducted in Cameroon [10] which indicated that only 9% of the study participants knew how to perform BSE. Furthermore, only 3 in 10 of the participants had overall good knowledge on BSE. This finding is higher than the results from Cameroon [10] and Yemen [14] which indicated that only 9.6% and 1.4% had good knowledge towards BSE, respectively.

In the present study, the main sources of information about BSE were mass media (39.8%) followed by health professionals (22.3%). Similarly, studies from Ethiopia [16], Nigeria [11], Cameroon [10], and Yemen [14] identified media as a leading source of information about BSE. However, the result from Malaysia indicated that printed media (brochure, newspapers, etc.) were main source of information. The result of the present study is an expected finding because, nowadays, most of young university students in our country are using Internet, television, and other mass media as a source of information. Therefore, use of these mass media would help increase awareness about BSE and breast cancer in general. Additionally, health professionals should also create awareness about BSE and breast cancer through health information dissemination and health education programs.

The result of multivariable logistic regression indicated that there was no significant relation between practice of BSE and the sociodemographic variables. However, participants who know how to perform BSE were 11 times, participants who know when to perform were 3.5 times, participants who know the three positions to perform were 2.3 times and participants who perceive BSE as important and useful were 6.8 times more likely to practice BSE as compared to those who do not know. This may be explained by the fact that knowledge of BSE was recognized as a necessary precursor to student's adherence performance of BSE [17].

Finding of this study should be interpreted with the following limitations. The study was cross-sectional, so causal conclusions cannot be drawn. The study was carried out in only students of Debre Berhan University and therefore might not be representative of other Universities of the country and young adults in general, and the practice of BSE may be different in other sectors of the population.

## 5. Conclusion

The results of the current study show that knowledge and practices of BSE among female DBU students were poor. Awareness on how to perform, when to perform, and position to perform BSE and perceiving BSE as important and useful to detect breast cancer were the predictor factors of practice of BSE among the female university students. The university and other stakeholders should develop educational programs that can increase knowledge and practice of breast self-examination among young female university students.

## Figures and Tables

**Table 1:** Sociodemographic characteristics of the study participants.

Variables	Category	Frequency	Percent
Age	≤19	52	13
	20–24	338	84.5
	≥25	10	2.5

College	Health science	62	15.5
	None health	338	84.5

Year of study	First year	50	12.5
	Second year	159	39.8
	Third year	83	20.8
	Four year	48	12
	Fifth year	60	15

Marital status	Single	240	60
	Single but in relationship	134	33.5
	Married	26	6.5

Family history of breast cancer	Yes	14	3.5
	No	386	96.5

**Table 2:** Knowledge about BSE among female DBU students.

Variables	Responses	Frequency	Percent
Ever heard of BSE	Yes	256	64
	No	144	36

Know how to perform BSE	Yes	143	35.8
	No	257	64.2

Know when to be performed	Yes	122	30.5
	No	278	69.5

Know the three positions to perform BSE	Yes	165	41.3
	No	235	58.7

Overall knowledge of BSE	Good	121	30.25
	Medium	130	32.5
	Poor	149	37.25

Sources of information on BSE	Mass media	130	39.8
	Health professions	73	22.3
	School	50	15.3
	Friends	40	12.2
	Family	17	5.2
	Others	17	5.2

**Table 3:** Attitude about BSE among female DBU students.

Attitudes	Response	Frequency	%
Know performing BSE if there is any risk factor	Yes	148	37
	No	252	63

BSE is important and useful to detect breast cancer	Agree	386	96.5
	Disagree	14	3.5

Early detection will increase the chance of long term survival	Agree	347	89.9
	Disagree	39	10.1

Where will you go, if there is any symptoms of breast cancer	Health facility	387	96.8
	Traditional healer	7	1.8
	Hide it	6	1.5

**Table 4:** Factors associated with practices of BSE among female DBU students.

Variables	Category	Practices of BSE		COR (95% CI)	AOR (95% CI)
		Yes (%)	No (%)		
College	Health science	76 (22.5)	262 (77.5)	**5.102 (2.891–9.004**)^**∗**^	1.158 (0.468–2.864)
	Nonhealth	37 (59.7)	25 (40.3)	1	1

Year of study	1st year	10 (20)	40 (80)	1	1
	2nd year	34 (21.4)	125 (78.6)	1.088 (0.494–2.397)	0.472 (0.142–1.565)
	3rd year	30 (36.1)	53 (63.9)	2.264 (0.992–5.167)	0.831 (0.222–3.108)
	4th year	20 (41.7)	28 (58.3)	**2.857 (1.162–7.025**)^**∗**^	0.881 (0.210–3.697)
	5th year	19 (31.7)	41 (68.3)	1.854 (0.768–4.473)	1.698 (0.463–6.231)

Ever heard of BSE	Yes	99 (38.7)	157 (61.3)	**5.855 (3.194–10.733**)^**∗**^	0.684 (0.278–1.686)
	No	14 (9.7)	130 (90.3)	1	1

Knowing how to perform BSE	Yes	97 (67.8)	46 (32.2)	**31.762 (17.159–58.793**)^**∗**^	**11.197 (4.542–27.607**)^**∗**^
	No	16 (6.2)	241 (93.8)	1	1

Knowing when to perform BSE	Yes	81 (66.4)	41 (33.6)	**15.187 (8.974–25.702**)^**∗**^	**3.507 (1.620–7.593**)^**∗**^
	No	32 (11.5)	246 (88.5)	1	1

Knowing the three positions to perform BSE	Yes	83 (50.3)	82 (49.7)	**6.917 (4.238–11.288**)^**∗**^	**2.253 (1.104–4.599**)^**∗**^
	No	30 (12.8)	205 (87.2)	1	1

Believing that BSE is important & useful to detect breast cancer	Agree	109 (31.4)	238 (68.6)	**5.496 (1.656–18.236**)^**∗**^	**6.837 (1.640–28.509**)^**∗**^
	Disagree	3 (7.7)	36 (92.3)	1	1

^*∗*^Statistical significance.

1: reference group.
